# Intra-islet glucagon signalling regulates beta-cell connectivity, first-phase insulin secretion and glucose homoeostasis

**DOI:** 10.1016/j.molmet.2024.101947

**Published:** 2024-04-26

**Authors:** K. Suba, Y. Patel, A. Martin-Alonso, B. Hansen, X. Xu, A. Roberts, M. Norton, P. Chung, J. Shrewsbury, R. Kwok, V. Kalogianni, S. Chen, X. Liu, K. Kalyviotis, G.A. Rutter, B. Jones, J. Minnion, B.M. Owen, P. Pantazis, W. Distaso, D.J. Drucker, T.M. Tan, S.R. Bloom, K.G. Murphy, V. Salem

**Affiliations:** 1 Department of Bioengineering, Imperial College London, London SW7 2AZ, United Kingdom; 2 Section of Investigative Medicine, Division of Diabetes, Endocrinology and Metabolism, Department of Metabolism, Digestion and Reproduction, Imperial College London, London W12 0NN, United Kingdom; 3 CHUM Research Center, University of Montreal, QC, Canada; 4 Section of Cell Biology and Functional Genomics, Division of Diabetes, Endocrinology and Metabolism, Department of Metabolism, Digestion and Reproduction, Imperial College London, London W12 0NN, United Kingdom; 5 Lee Kong Chian Imperial Medical School, Nanyang Technological University, Singapore; 6 Imperial College Business School, Imperial College London, London SW7 2AZ, United Kingdom; 7 Lunenfeld-Tanenbaum Research Institute, Mount Sinai Hospital, University of Toronto, Toronto, Canada

**Keywords:** Islet calcium imaging, Calcium waves, Glucagon

## Abstract

**Objective:**

Type 2 diabetes (T2D) is characterised by the loss of first-phase insulin secretion. We studied mice with β-cell selective loss of the glucagon receptor (Gcgr^*fl/fl*^ X Ins-1^*Cre*^), to investigate the role of intra-islet glucagon receptor (GCGR) signalling on pan-islet [Ca^2+^]_I_ activity and insulin secretion.

**Methods:**

Metabolic profiling was conducted on Gcgr^*β-cell−/−*^ and littermate controls. Crossing with GCaMP6f (STOP flox) animals further allowed for β-cell specific expression of a fluorescent calcium indicator. These islets were functionally imaged *in vitro* and *in vivo*. Wild-type mice were transplanted with islets expressing GCaMP6f in β-cells into the anterior eye chamber and placed on a high fat diet. Part of the cohort received a glucagon analogue (GCG-analogue) for 40 days and the control group were fed to achieve weight matching. Calcium imaging was performed regularly during the development of hyperglycaemia and in response to GCG-analogue treatment.

**Results:**

Gcgr^*β-cell−/−*^ mice exhibited higher glucose levels following intraperitoneal glucose challenge (control 12.7 mmol/L ± 0.6 vs. Gcgr^*β-cell−/−*^ 15.4 mmol/L ± 0.0 at 15 min, *p* = 0.002); fasting glycaemia was not different to controls. *In vitro*, Gcgr^*β-cell−/−*^ islets showed profound loss of pan-islet [Ca^2+^]_I_ waves in response to glucose which was only partially rescued *in vivo*. Diet induced obesity and hyperglycaemia also resulted in a loss of co-ordinated [Ca^2+^]_I_ waves in transplanted islets. This was reversed with GCG-analogue treatment, independently of weight-loss (n = 8).

**Conclusion:**

These data provide novel evidence for the role of intra-islet GCGR signalling in sustaining synchronised [Ca^2+^]_I_ waves and support a possible therapeutic role for glucagonergic agents to restore the insulin secretory capacity lost in T2D.

## Highlights

• Beta-cell selective loss of the glucagon receptor (Gcgr^*β-cell−/−*^) results in defective insulin secretion.
• Gcgr^*β-cell−/−*^ islets display reduced ability for calcium waves to propagate and reduced beta-cell connectivity.
• Long-term glucagon therapy reverts the loss of beta-cell calcium waves and dysglycaemia associated with diet induced obesity.

## 1 Introduction

The earliest hallmark of type 2 diabetes (T2D) is the loss of first-phase insulin secretion [1]. More recently it is recognised that the disordered insulin secretion that occurs in T2D is associated with altered functional connectivity between β-cells within the islet [2,3]. Insulin secretion is closely related to islet-wide [Ca^2+^]_I_ oscillatory activity [4] and there is increasing attention turned towards understanding the islet of Langerhans as a functional unit. β-cell heterogeneity, specifically subpopulations of β-cell hubs or first responders [[5], [6], [7], [8]], are thought to be important in the control of coordinated insulin release from pancreatic islets. However, electrical coupling alone, via ionic gap junctions, is not enough to explain the observed connectivity patterns between β-cells [9], which prompted us to look at the modulatory role of paracrine factors, in particular α-cell-derived glucagon.

Glucagon is typically described as a counter-regulatory hormone to insulin, acting in the fasting state to raise blood glucose via hepatic glycogenolysis and gluconeogenesis. However, glucagon signalling has pleiotropic effects which are increasingly recognised, and its role in diabetes remains to be fully elucidated [10]. The insulinotropic effects of glucagon have been known for many years [11]. Glucagon infusions in healthy [12] and non-obese subjects with T2D [13] result in a rapid and substantial increase in plasma insulin levels under hyperglycaemic conditions. With the recent focus on targeting the glucagon receptor to treat diabetes, the role of intra-islet glucagon signalling is of increasing interest. Most of our understanding of the intra-islet effects of glucagon has come from *in vitro* or *ex vivo* studies [14,15] using isolated islets and high concentrations of glucagon. These corroborate the insulinotropic effects of glucagon, but attribute them, at least in part, to β-cell GLP-1 receptor activation [[16], [17], [18], [19]], since intra-islet levels of glucagon may be higher than systemic levels and glucagon can activate the GLP-1 receptor at high concentrations [20]. Glucagon can have direct effects on multiple individual β-cells, likely via its effects on intracellular cAMP signalling, but this may also act to modulate how cells in the pancreatic islet are coupled electrically, potentially influencing how their activity is coordinated. Overall, it remains poorly understood how intra-islet glucagon augments β-cell function, over what dynamic range, and the physiological and pharmacological relevance of intra-islet glucagon receptor activation.

We aimed to characterise the effects of β-cell selective loss of the glucagon receptor (GCGR) on islet connectivity and insulin secretion. We hypothesised that intra-islet GCGR signalling contributes to the maintenance of islet-wide β-cell functional connectivity and normal insulin secretion. We then examined whether glucagon receptor agonist therapy in the setting of obesity-induced dysglycaemia directly restores islet β-cell functional connectivity.

## 2 Results

### 2.1 Gcgr^*β-cell−/−*^ islets have normal expression levels of β-cell identity and “disallowed” genes, but display disturbed insulin secretion profiles

**Figure 1:**
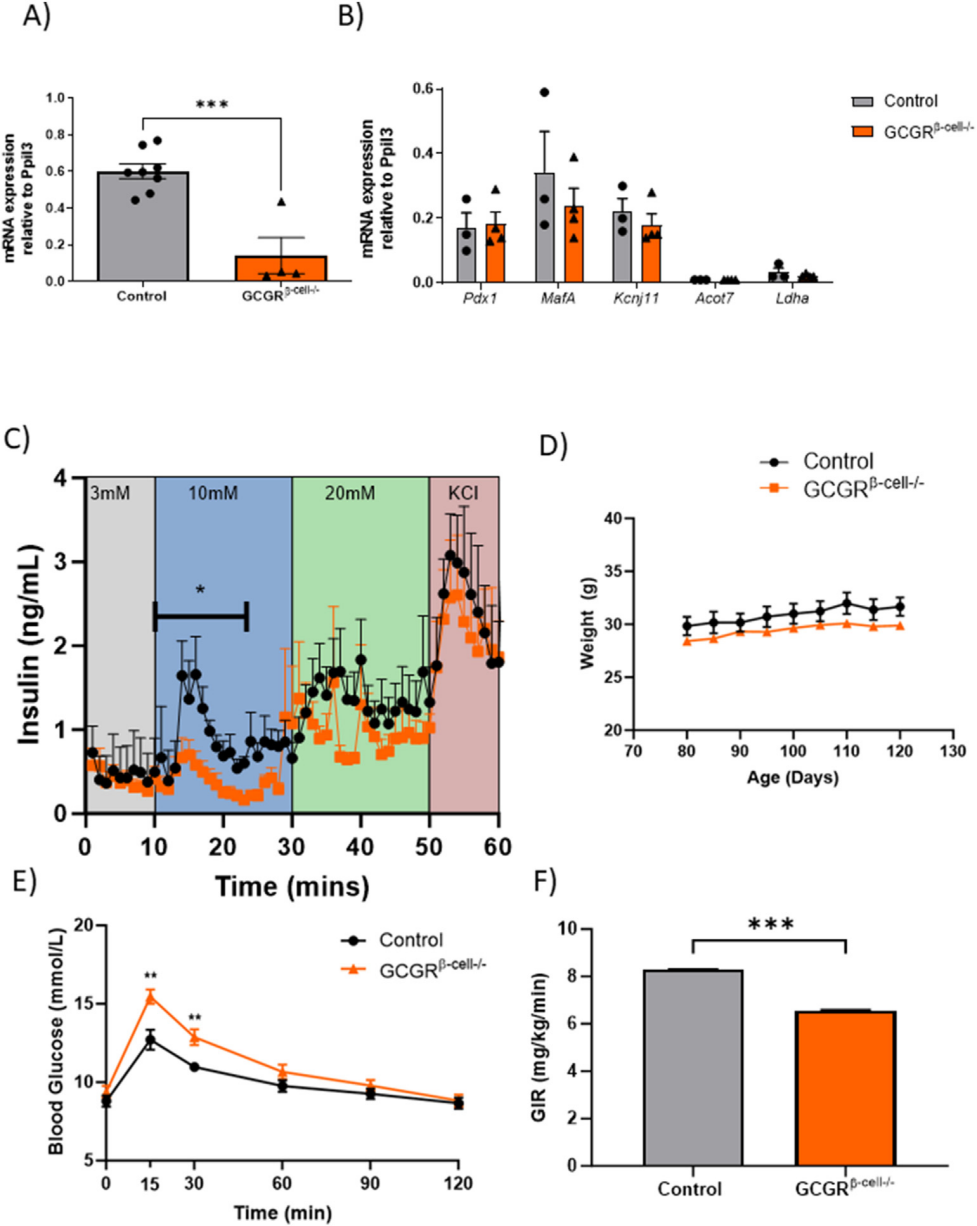
**The β-cell specific deletion of the glucagon receptor results in impaired insulin secretion *ex vivo* and *in vivo***. A) The expression of GCGR relative to *Ppil3* is reduced from in Gcgr^*β-cell−/−*^ islets (unpaired t-test n = 4 animals per genotype, *p* = 0.0187), while B) the relative expression of β-cell identity genes *Pdx1, Mafa, Kcnj11, Acot7* and *Ldha* is not altered in Gcgr^*β-cell−/−*^ islets (n = 3) versus controls (n = 4; unpaired t-tests; *p* = ns). C) *In vitro* perifusion assay to assess glucose-stimulated insulin responses of control (n = 50IEQ per experiment; 200 islets from 3mice) and Gcgr^*β-cell−/−*^ islets (n = 50IEQ per experiment; 300 islets from 4 mice) shows that insulin secretion is decreased in Gcgr^*β-cell−/−*^ islets between 10 min. and 25 min, when islets are stimulated with 10 mM glucose (Unpaired t-test; *p* = 0.048). D) The body weight of 12-week old Gcgr^*β-cell−/−*^animals (n = 15) is comparable to littermate controls (n = 10). E) Gcgr^*β-cell−/−*^ animals show significantly increased blood glucose concentrations at 15 min. and 30 min. time-points in an IPGTT (control vs Gcgr^*β-cell−/−*^, t = 15, 12.7 mmol/L ± 0.6, 15.4 mmol/L ± 0.5; t = 30, 11.0 mmol/L ± 0.3, 12.9 mmol/L ± 0.5; control n = 9; Gcgr^*β-cell−/−*^ n = 15; Two-tailed, Unpaired t-test; *p* = 0.002). F) Hyperglycaemic (blood glucose: 16.5 mmol/L ± 1.5) clamp experiments revealed a lower glucose infusion rate in Gcgr^*β-cell−/−*^ animals compared with age-matched controls (AUC of 66 mg/kg/min ± 0.3 versus 83 mg/kg/min ± 0.1 for Gcgr^*β-cell−/−*^ versus control animals, respectively; Gcgr^*β-cell−/−*^ n = 5, control n = 2; Two-tailed, Unpaired t-test on AUC; ∗∗∗*p* < 0.001).

Mice on a C57BL/6J background were bred to knock out the glucagon receptor (GCGR) selectively in β-cells (Gcgr^*β-cell−/−*^) using the model of Gcgr^*fl/fl*^ crossed with an Ins1^*Cre*^ line (see Methods for full details). The islets of these mice were isolated at 12 weeks of age. Quantification of mouse GCGR mRNA using qPCR on whole islets revealed a large reduction in GCGR expression in Gcgr^*β-cell−/−*^ animals versus littermate controls (Figure 1A relative expression 0.55 ± 0.04 for control vs 0.14 ± 0.10 for Gcgr^*β-cell−/−*^; *p* = 0.02). There was no evidence of compensatory upregulation of GLP-1 or GIP receptor expression levels in Gcgr^*β-cels−/−*^ islets (Supplementary Figure 1A and B). The anatomy of Gcgr^*β-cell−/−*^ islets was unperturbed, with no differences in relative β-cell mass (β- to α-cell ratio) when compared with littermate controls (Supplementary Figure 1C–E). mRNA expression of established β-cell identity markers (*Pdx1, Mafa, Kcnj11*) and “disallowed” (*Acot7, Ldha*) genes [21] were also comparable to control levels (Figure 1B). Taken together, these experiments suggest that germline Ins1^*Cre*^-driven deletion of GCGR in β-cells is not associated with changes in islet maturation or architecture. However, standard *exvivo* perifusion experiments revealed that insulin secretion as glucose was first elevated from 3 to 10 mM, was diminished in Gcgr^*β-cell−/−*^ versus WT islets (n = 200–300 islets from 3 to 4 animals per phenotype). Whilst the response to 20 mM glucose after 30 min of perifusion and the final addition of KCl did not elicit detectably different insulin secretion between phenotypes, the AUC for insulin between 10 and 25 min of the experiment (the 10 mM glucose phase) was 5.4 (95% CI 3.8– 6.9) for Gcgr^*β-cell−/−*^ versus 13.0 (95% CI 10.1–15.8) for the WT islets (*p* = 0.048) (Figure 1C).

### 2.2 Gcgr^*β-cell−/−*^ mice exhibit an insulin secretory deficit

Unlike mice with a whole body deletion of the glucagon receptor [22], our model of β-cell selective loss of the glucagon receptor did not result in a body weight phenotype: adult body weight at 12 weeks was 31.7 ± 0.9 g for controls (n = 10) versus 29.9 ± 0.9 g for Gcgr^*β-cell−/−*^animals (n = 15) (*p* = ns; Figure 1D). Circulating glucose levels following 5 h fasting were similar in Gcgr^*β-cell−/−*^mice and controls, but during an intraperitoneal glucose tolerance test (IPGTT), circulating glucose concentrations were significantly higher at 15 min and 30 min following glucose in Gcgr^*β-cell−/−*^animals (Figure 1E). Insulin tolerance (ITT) and hepatic glucose mobilisation (as measured with a pyruvate tolerance test) were not different in Gcgr^*β-cell−/−*^animals (Supplementary Figure 2A and B), and basal levels of circulating insulin, glucagon and GLP-1 were also similar to controls (Supplementary Figure 3A–D). In this context, the reduced glucose tolerance suggested impaired glucose-stimulated insulin secretion (GSIS) in Gcgr^*β-cell−/−*^ animals. To further test this, we conducted hyperglycaemic clamp experiments on Gcgr^*β-cell−/−*^ animals and littermate controls (Figure 1F and Supplementary Fig. 4). We found that the glucose infusion rate (GIR) was significantly lower in the (weight-matched) Gcgr^*β-cell−/−*^cohort (66 mg/kg/min ± 0.3 versus 83 mg/kg/min ± 0.1 for controls; *p* = 0.001). Taken together, these experiments suggest that β-cell loss of GCGR signalling results in impaired early GSIS.

### 2.3 *In vitro* calcium imaging reveals that β-cell GCGR loss affects coordinated [Ca^2+^]_I_ activity

Islet (β-cell) intracellular calcium [Ca^2+^]_I_ oscillations underpin insulin secretion [23]. To better understand the mechanisms driving the insulin secretory deficit in Gcgr^*β-cell−/−*^ mice, these animals were further crossed with a line expressing the [Ca^2+^]_I_ indicator GCaMP6f selectively in the β-cells. *In vitro*, Ins1^*Cre*^GCaMP6f^*fl/fl*^: Gcgr^*β-cell−/−*^ and control (Ins1^*Cre*^GCaMP6f^*fl/fl*^: Gcgr^*β-cell+/+*^) islets were calcium imaged across an increasing glucose concentration on a perifusion stage.

**Figure 2:**
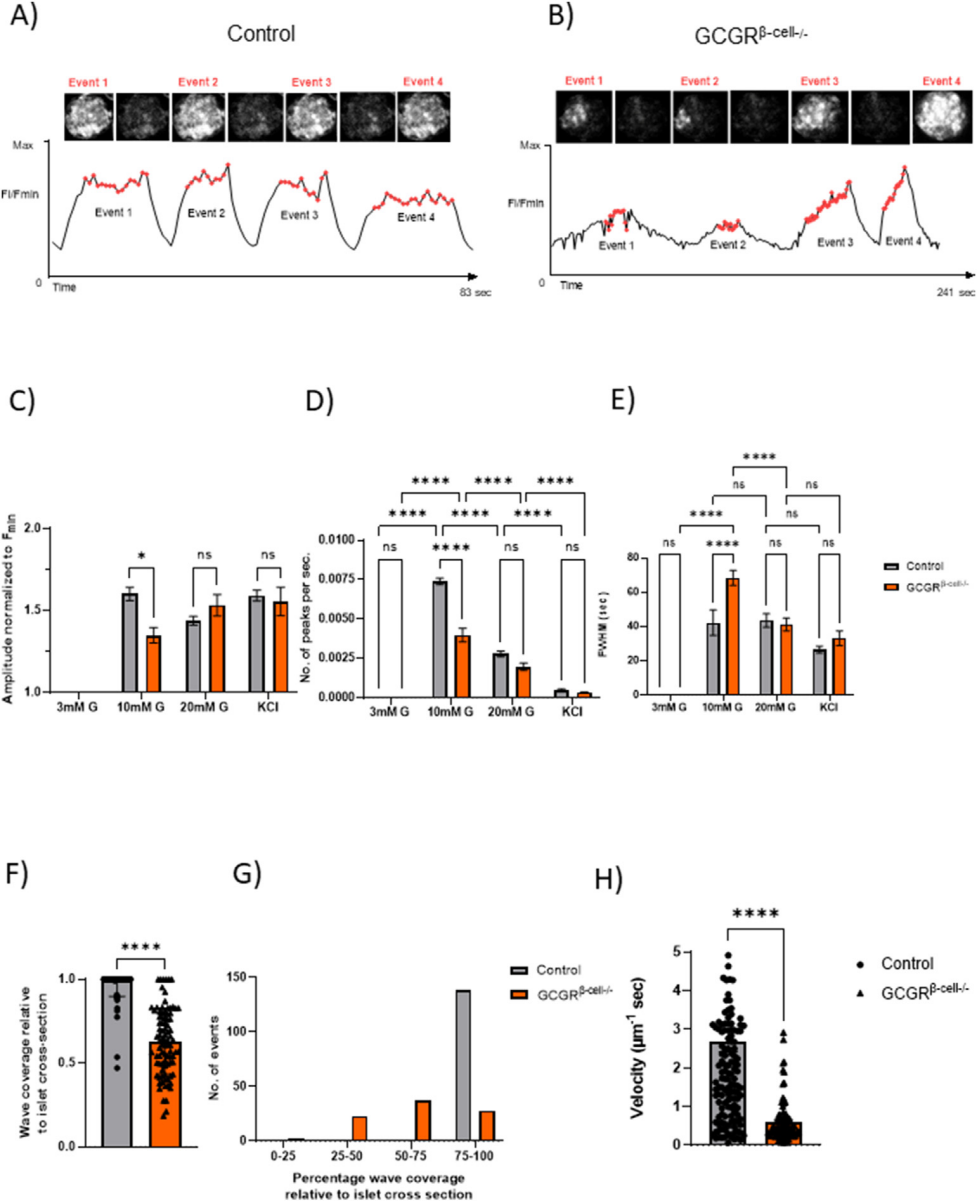
**Gcgr^*β-cell−/−*^ islets show altered [Ca^2+^]_I_ dynamics in *vitro***. Representative images and [Ca^2+^]_I_ traces of most commonly observed wave patterns in GCaMP-expressing control (Ins1^*Cre*^GCaMP6f^*fl/fl*^) (A) and GCGR^*β-cell−/−*^(Ins1^*Cre*^GCaMP6f^*fl/fl*^: Gcgr^*β-cell−/−*^) (B) islets at 10 mM glucose concentration. C) The normalised amplitude of [Ca^2+^]_I_ waves was significantly smaller in GCGR^*β-cell−/−*^ islets (n = 81 islets from 3 animals) compared to control (n = 63 islets from 4 animals) values at 10 mM glucose concentration (Two-way ANOVA; Sidak's test; *p* = 0.0147). D) Wave events occurred at a significantly lower frequency in Gcgr^*β-cell−/−*^ islets compared to control islets at 10 mM glucose levels (∗∗∗∗*p* < 0.0001) E) but activity lasted longer, as reflected by the increased full-width at half maximum (FWHM) values at the same glucose concentration (Two-way ANOVA; Tukey's and Sidak's test; ∗∗∗∗*p* < 0.0001). F) At 10 mM glucose, wave events extended over the entire islet cross-section in control islets (n = 140 events from 45 islets) whereas in Gcgr^*β-cell−/−*^ islets (n = 90 events from 45 islets), a significantly smaller area of the islet cross-section was involved in waving activity (Unpaired t-test ∗∗∗∗*p* < 0.0001). G) Binning all wave events on the basis of the proportion of islet cross-section covered revealed that out of the total of 90 events encountered in Gcgr^*β-cell−/−*^ islets, 23 events extended to 25–50% of the islet cross-section, 37 events extended to 50–75% of the islet-cross-section and 28 events involved 75–100% of the islet cross-section. In control islets, the overwhelming majority of events (138 out of 140) extended over the 75–100% of the islet cross-section. H) The velocity of wave events (regardless of the proportion of islet cross-section covered) was significantly decreased in Gcgr^*β-cell−/−*^ islets (∗∗∗∗*p* < 0.0001; Mann–Whitney test).

There were visually apparent differences in the tendency for β-cells to fluoresce in a co-ordinated fashion and propagate [Ca^2+^]_I_ “waves” across the control compared with the Gcgr^*β-cell−/−*^ islets (Supplementary Videos 1 and 2). Figure 2A displays a typical run of [Ca^2+^]_I_ “waves” spanning the entire control islet cross section at 10 mM glucose. At this glucose concentration, synchronised [Ca^2+^]_I_ oscillations, spanning the entire islet cross-section, were evident in all control islets (n = 63 islets from 4 mice). In contrast, localised β-cell activity in the Ins1^*Cre*^GCaMP6f^*fl/fl*^: Gcgr^*β-cell−/−*^islets (n = 81 islets from 3 animals) much more rarely resulted in waves that propagated across the whole islet cross section. Whole islet analysis of this [Ca^2+^]_I_ activity suggested that these behaviours were most pronounced at the start of the perifusion experiments, as the glucose level rose from 3 mM to 10 mM (Figure 2C–E). Given that [Ca^2+^]_I_“waves” were less likely to be pan-islet in the Ins1^*Cre*^GCaMP6f^*fl/fl*^: Gcgr^*β-cell−/−*^islets, it follows that the amplitude of the waves was lower (Figure 2C). Also at 10 mM glucose, the frequency of waves across the Gcgr^*β-cell−/−*^islets was lower (Figure 2D) with a broader FWHM (Figure 2E). These data are alternatively represented in Figure 2Fand G as the proportion of the total islet cross section covered by any given wave (90–140 events per group were analysed). Overall, a significantly smaller proportion of the entire islet cross section was covered by waves in the Gcgr^*β-cell−/−*^islets (Figure 2F). Whilst all of the waves imaged propagated across >75% of the control islets at 10 mM glucose, the waves across the Gcgr^*β-cell−/−*^islets were just as likely to cover <75% or <50% of an islet cross section (Figure 2G). As might be anticipated from the observation that the waves in the Gcgr^*β-cell−/−*^islets were less frequent and had a broader FWHM, the velocity of the waves analysed in the Gcgr^*β-cell−/−*^islets were significantly slower than in controls (Figure 2H).

Supplementary video related to this article can be found at https://doi.org/10.1016/j.molmet.2024.101947

The following are the supplementary data related to this article:

Supplementary Video 1 and 2. Representative recordings of [Ca^2+^]_I_ activity in control (1) and Gcgr^*β-cell−/−*^ islets (2) at 10 mM glucose concentration. Videos were edited for visualisation purposes on FIJI and Adobe Premiere Pro, the elapsed time is shown in MM:SS format.

**Figure 3:**
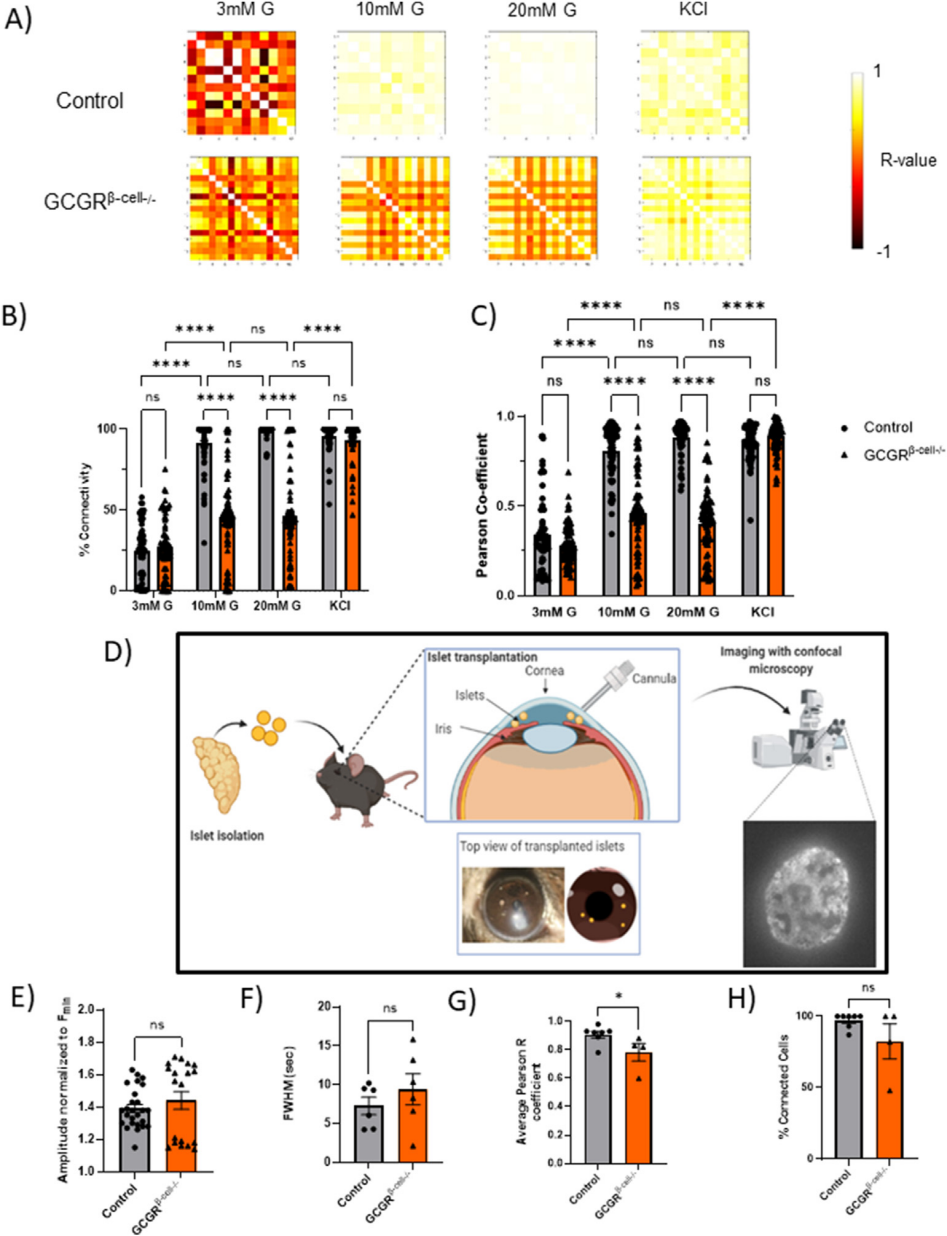
**β-Cell functional connectivity is reduced in GCGR^*β-cell−/−*^ islets and this is partially restored following implantation in the murine eye**. A) Subjecting the [Ca^2+^]_I_ traces of individual β-cells to connectivity analysis (previously described in Salem et al., 2019) reveals a reduction in functional connectivity in Gcgr^*β-cell−/−*^ islets. Both percentage connectivity (B) and average Pearson R values (C) are significantly lower at 10 and 20 mM glucose levels in Gcgr^*β-cell−/−*^ islets (Pearson R values: 0.81 ± 0.02 for control versus 0.46 ± 0.02 for GCGR^*β-cell−/−*^ islets at 10 mM; Two-way ANOVA; Tukey's test *p* < 0.0001), while they remain comparable to control levels when stimulated with KCl. D) Schematic of the experimental imaging platform. Islets with β-cell specific loss of the GCGR that also express the GCaMP6f [Ca^2+^]_I_ reporter (Ins1^*Cre*^GCaMP6f^*fl/fl*^: Gcgr^*β-cell−/−*^-islets) are isolated and transplanted into the anterior camber of the eye of a syngeneic recipient. Ins1^*Cre*^GCaMP6f^*β-cell+/+*^-islets were implanted as a control. After 4 weeks, the islets implant, are innervated and functional. Thereafter they can be directly imaged with confocal fluorescent microscopy longitudinally. A total of 7 Ins1^*Cre*^GCaMP6f^*fl/fl*^: Gcgr^*β-cell−/−*^-islets and 8 Ins1^*Cre*^GCaMP6f^*β-cell+/+*^-islets were imaged under high circulating glucose conditions (in three animals). Seven control islets and only four Ins1^*Cre*^GCaMP6f^*fl/fl*^: Gcgr^*β-cell−/−*^ islets exhibited [Ca^2+^]_I_ waves. E) The amplitude and (F) FWHM values of [Ca^2+^]_I_ activity were not significantly different between control and Gcgr^*β-cell−/−*^-islets. Connectivity analysis of single cell read-outs showed that (G) the average coefficient of connectivity was significantly lower in islets where the GCGR was deleted in β-cells specifically (Two-tailed, Mann–Whitney test; *p* = 0.036). H) The average number of connected β-cells was lower but this effect was not significant (Two-tailed, Mann–Whitney test; *p* = ns).

We then analysed single cell [Ca^2+^]_i_ dynamics in the waving islets, applying statistical methods to measure correlations between β-cell [Ca^2+^]_I_ activity, using previously described methods [6]. Driven by the lack of ability of [Ca^2+^]_I_waves to propagate fully, all indices of connectivity were statistically lower in Gcgr^*β-cell−/−*^islets at 10 and 20 mM glucose. The addition of 40 mM KCl to Gcgr^*β-cell−/−*^ islets elicited a strong pan-islet [Ca^2+^]_I_ response, suggesting that a powerful depolarising stimulus is able to overcome the defective communication between β-cells (Figure 3A–C). Taken together, these observations suggest that β-cell GCGR signalling is important for the generation of pan islet [Ca^2+^]_I_ waves in response to glucose *in vitro*. Defective β-cell communication and the lack of synchronisation may partially explain the insulin secretory deficit noted in Gcgr^*β-cell−/−*^ animals.

### 2.4 *In vivo* [Ca^2+^]_I_ imaging reveals that the loss of co-ordinated calcium activity in Gcgr^*β-cell−/−*^ islets is only partially rescued *in vivo*

*In vitro* perifusion studies cannot recapitulate the complex microenvironment of *in situ* islets, where their activity may be modulated by neuronal input and a capillary bed. To directly investigate β-cell connectivity of Gcgr^*β-cell−/−*^ *in vivo*, Ins1^*Cre*^GCaMP6f^*fl/fl*^: Gcgr^*β-cell−/−*^-expressing islets were implanted into the eyes of syngeneic WT recipients, where they act as faithful “reporters” that can be directly imaged [24] (control: n = 8 islets in 3 recipients; Gcgr^*β-cell−/−*^: n = 7 islets in 3 recipients; Figure 3D). Following full implantation, islet [Ca^2+^]_I_ dynamics were recorded under high circulating glucose (10.4 ± 1.5 mmol/L and 11.3 ± 0.4 mmol/L for Ins1^*Cre*^GCaMP6f^*fl/fl*^: Gcgr^*β-cell−/−*^and control groups respectively; *p* = ns). In the islets from control animals, 7 out of 8 islets were observed to have cross-sectional [Ca^2+^]_I_ wave activity as opposed to only 4 out of 7 of the Gcgr^*β-cell−/−*^ islets. The amplitude of the waves in the Gcgr^*β-cell−/−*^ islets were not measurably different and there was only a tendency for their FWHM to be broader (slower wavefront) as was detected *in vitro* (Figure 3E and F). The average Pearson R values of Ins1^*Cre*^GCaMP6f^*fl/fl*^: Gcgr^*β-cell−/−*^islets was significantly lower than in the control islets (R = 0.88 ± 0.06 compared to R = 0.98 ± 0.02, *p* = 0.036) (Figure 3G). In parallel, the average proportion of connected cells was non-significantly lower in islets lacking β-cell GCGR(82.1 ± 12.2% compared to 97.1 ± 1.96% for control islets; *p* = ns) (Figure 3H). We conclude that the detrimental effects of GCGR knockout on β-cell [Ca^2+^]_I_ dynamics are only partially rescued post implantation *in vivo*.

### 2.5 Diet-induced obesity (DIO) results in reduced β-cell connectivity which can be restored by chronic administration of a GCG-analogue

**Figure 4:**
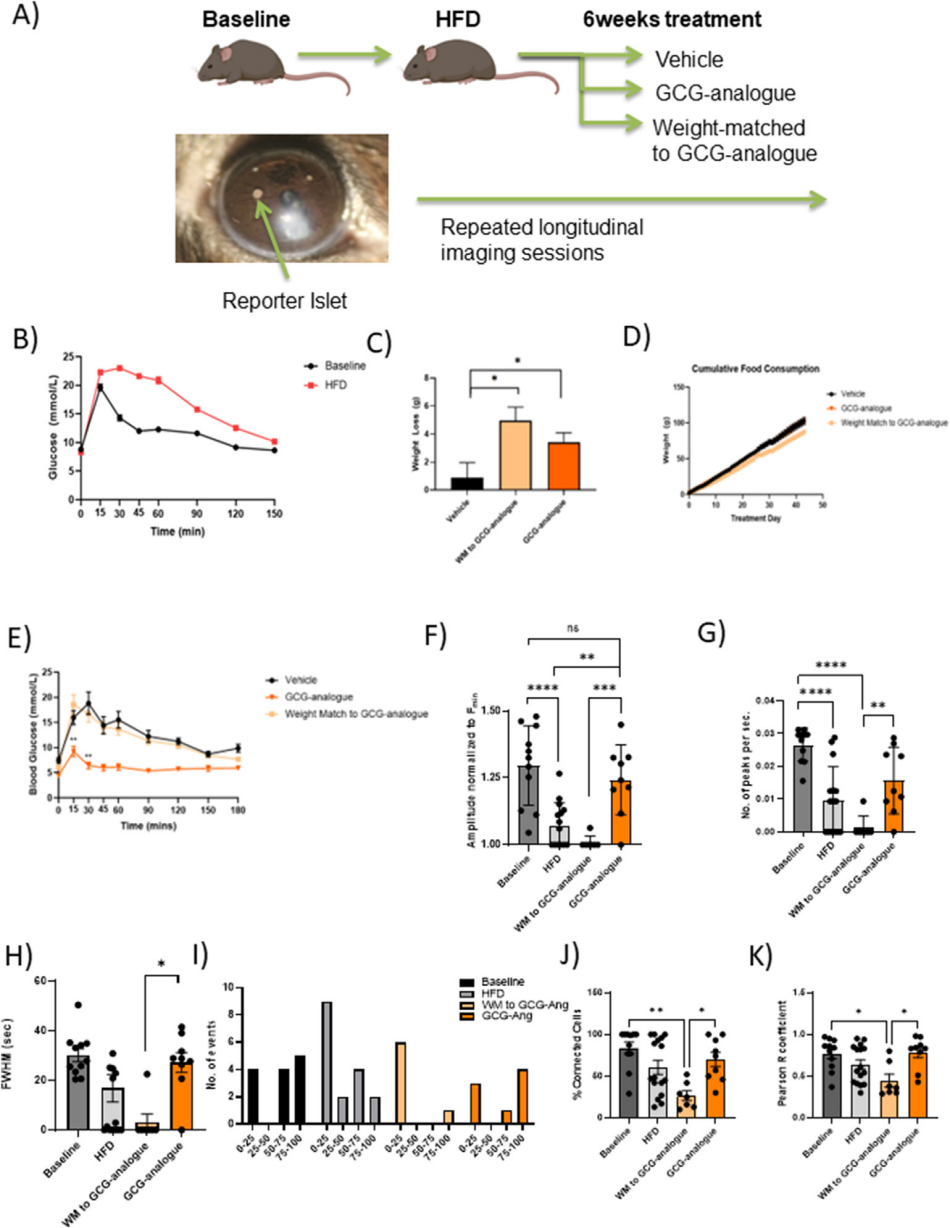
**Direct observation of islet function following the induction of diet-induced obesity in mice and subsequent treatment with a synthetic glucagon-analogue (GCG-analogue)**. A) Schematic diagram of the study. B) High-fat feeding of 10-week old mice leads to the development of diet-induced obesity (DIO) and impaired glucose tolerance (n = 63–59; the AUC at baseline was 508.3 ± 106 versus 1309 ± 154.9 after HFD; Two-tailed Welch's t-test; ∗∗∗∗*p* < 0.0001). C) The mean bodyweight was 39.6 ± 0.9 g prior to the start of the treatment and was comparable across groups. GCG-analogue treatment caused a significant weight-loss of −12.01% ± 1.97 versus −2.3% ± 2.7 in the vehicle group (n = 8 per group treatment group, one-way ANOVA, Bonferroni multiple comparison test; *p* = 0.029). D) Cumulative food consumption over the course of the study was comparable between the three treatment groups (one-way ANOVA, Tukey's test; *p* = ns). E) GCG-analogue treatment improves glycaemia of DIO mice. Glucose concentrations at the 15 min and 30 min time-points of an IPGTT were significantly lower in the GCG-analogue group compared to the weight-matched group (t15 = 9.2 mmol/L ± 1 versus 12.5 mmol/L ± 2 for GCG-analogue versus WM to GCG-analogue groups *p* = 0.001; t30 = 6.4 mmol/L ± 0.7 versus 16.7 mmol/L ± 1.6 for GCG-analogue versus WM to GCG-analogue groups *p* = 0.001; One-way ANOVA; Tukey's test). F) Longitudinal imaging of reporter islets implanted in the anterior eye chamber revealed that with high-fat feeding, the normalised amplitude of [Ca^2+^]_I_ is significantly reduced from baseline (One-way ANOVA; Tukey's test; ∗∗∗∗*p* < 0.0001). Treatment with the GCG-analogue restored the amplitude of wave events to baseline values (*p* = ns). Similarly, the frequency (G) as well as the wavelength (H) of wave events decreased with high-fat feeding, a trend that was reversed with the administration of the GCG-analogue but not weight-loss (frequency: WM to GCG-analogue versus GCG-analogue groups; *p* = 0.007; wavelength: WM to GCG-analogue versus GCG-analogue groups; *p* = 0.017; One-way ANOVA; Tukey's test). Of note, a significant number of islets in the HFD (6 out of 15 islets) and WM to GCG-analogue (6 out of 7 islets) groups showed no [Ca^2+^]_I_ ocillations over the observed period and these datapoints are represented as 0 on FWHM and frequency graphs. I) Dysglycaemia (which occurred with high-fat diet) was associated with a reduction in the percentage of islet cross-section involved in wave activity of reporter islets. This trend was reversed with GCG-analogue treatment when 4 out of 8 islets displayed wave events which involved 75–100% of the islet cross-section. J) and K) HFD results in reduced β-cell connectivity and this is rescued by the GCG-analogue (Baseline n = 11 islets; HFD n = 16 islets; WM to GCG-analogue n = 7 islets; GCG-analogue n = 9 islets; Percentage connectivity: Kruskal–Wallis test; *p* = 0.015; Pearson R values: Kruskal–Wallis test; *p* = 0.045).

Selective deletion of the GCGR in β-cells revealed a possible beneficial physiological role for GCGR signalling in islet functional connectivity. Consequently we investigated whether pharmacological GCGR agonism in a diet-induced obese model of chronic hyperglycaemia can alleviate the deleterious effects of high-fat diet on co-ordinated insulin secretion (Figure 4A). We created a glucagon analogue (GCG-analogue, see Methods and Supplementary Figure 5 for sequence) with strong potency for cAMP production at the mouse glucagon receptor (m GCGR) but not different than native glucagon at the mGLP-1R. The analogue was 164 times more potent at the mouse glucagon compared to the mouse GLP-1 receptor (m GCG > mGLP-1R: ΔΔpEC_50_ = 2.2 ± 0.3). In static incubation it significantly enhanced glucose stimulated insulin secretion from mouse islets over ten-fold (Supplementary Figure 6).

Twenty-four lean C57BL/6J mice (aged 8 weeks) received syngeneic transplants of Ins1^*Cre*^GCaMP6f^*fl/fl*^-expressing “reporter” islets into their anterior eye chamber and were then placed on a high fat diet (HFD). Following 12 weeks of the HFD, the mice demonstrated diet-induced obesity (DIO) and impaired glucose tolerance (Figure 4B). Animals were then randomised by body weight into three groups of 8: Group 1 received a daily dose of GCG-analogue, Group 2 were control/vehicle-injected and fed *ad libitum* and Group 3 received daily control/vehicle injections but were food restricted to weight match (WM) to Group 1. Forty days of GCG-analogue treatment resulted in a body weight loss in Group 1 of 12.01% ± 1.97 (*p* = 0.030; Figure 4C). The cumulative food intake in Group 3 was significantly less than both Groups 1 and 2, supporting previous reports of an increased energy expenditure mechanism for weight loss with exogenous administration of a glucagon agonist [25] (Figure 4D).

Treatment with the GCG-analogue produced a weight loss independent normalisation of glucose homoeostasis in DIO animals (Figure 4E). Fasting glucose was lower (4.3 mmol/L versus 7.5 mmol/L; *p* = 0.005) and intraperitoneal glucose tolerance was improved (peak glucose of 9.2 mmol/L versus 18.5 mmol/L) in GCG-analogue versus weight matched-groups, respectively (*p* < 0.001).

In order to understand the direct islet effects of GCG-analogue, we monitored the activity of the reporter islets in these animals across the duration of the study. Firstly we demonstrate longitudinally and *in vivo*, a disruption in β-cell coordinated [Ca^2+^]_I_ activity related to obesity. After 3 months on a HFD (when obese and dysglycaemic), fewer reporter islets were waving and when they did the amplitude and frequency of the [Ca^2+^]_I_ waves was reduced (Figure 4F–H). There was also a tendency for those waves to propagate over a smaller proportion of the islet cross section (Figure 4I), as occurred in the Gcgr^*β-cell−/−*^ islets *in vitro*. The worsening glycaemic phenotype of DIO mice was parallelled by a diminution of pan-islet β-cell connectivity that was recovered by GCG-analogue treatment but not weight loss alone (Figure 4J and K). Of note, blood glucose levels throughout the imaging sessions were comparable (10.6 ± 0.52 mmol/L versus 12.7 ± 0.6 mmol/L versus 10 ± 0.3 mmol/L over three imaging sessions), therefore differences in [Ca^2+^]_I_ responsiveness were unlikely to have been a consequence of low circulating glucose levels during the imaging studies. Taken together, our findings indicate that DIO impairs coordinated [Ca^2+^]_I_ responses of β-cells and that these coordinated responses can be restored by GCG-analogue treatment via mechanisms that are independent of weight loss.

## 3 Discussion

This study investigated the physiological role of intra-islet glucagon signalling and the pharmacological effects of glucagon receptor activation in islets. We found that β-cell selective deletion of the GCGR results in a glucose intolerant phenotype that is redolent of early T2D, with a loss of first-phase insulin secretion. We also provide evidence of the utility of GCGR-preferring peptide analogues for the recovery of obesity-induced dysglycaemia.

Our model of β-cell selective GCGR deletion resulted in a large reduction in whole-islet measured Gcgr transcript, consistent with reports that most of the GCGR in the islet resides on β-cells [26]. The glucose intolerant phenotype in these mice was associated with an insulin secretory deficit without any changes to islet architecture or β-cell mass. Furthermore, the intra-islet receptor expression and serum levels of two major incretins GLP-1 and GIP, were not different from controls, suggesting a lack of compensatory morphological or functional changes that are common in global enteropancreatic-hormone receptor knockout models. Zhang et al. recently reported a glucose intolerant phenotype in their model of β-cell GCGR loss although they did not report on [Ca^2+^]_I_ dynamics [27]. Conversely, others who have studied intra-islet GCGR signalling in germline [28] or conditional [15] β-cell knock out of the GCGR using a mouse insulin promoter (MIP)^*Cre*^-driven mechanism have not reported glucose intolerance. This may be due to differences in promoter activity. *Ins1* promoter-driven systems, and particularly the Ins-1^*Cre*^ knock-in model used here, are highly specific to β-cells [29,30], with no reported extra-pancreatic expression or alterations to glucose homoeostasis. Differences in the animal models used to investigate islet paracrinology must be taken into account when comparing studies [31]. Whilst there was no measurable difference on GLP-1R or GIPR expression in our Gcgr^*β-cell−/−*^ islets, there remains the possibility of other more subtle reorganisation of GPCR signalling following receptor deletion.

We propose that the highly selective loss of the GCGR on β-cells in mice results in a phenotype that resembles some of the earliest manifestations of T2D. Here we show that first phase insulin secretion seems to be most affected with this phenotype since by 120 min on the IPGTT tests glucose levels had indeed returned to normal, whilst the insulin secretory defect on *in vitro* perifusion was only seen during the first rise from 3 to 10 mM glucose. Fascinating work by Nunemaker et al. revealed that the shape of the individual secretory bursts from individual islets and from *in vivo* sampling are almost superimposable (corrected for amplitude), suggesting that the *in vivo* pulse is generated by simultaneous secretion from an imprinted islet population [4]. Therefore, understanding the islet-level structure/function relationships that sub-serve insulin secretion is important [32].

*In vitro*, Gcgr^*β-cell−/−*^ islets possessed β-cells that were able to respond to a rise in glucose with insulin secretion. However, this response was impaired and notably all groups of activated β-cells within the Gcgr^*β-cell−/−*^ islets seemed much less able to propagate that [Ca^2+^]_I_ wave across the entire islet structure. [Ca^2+^]_I_ waves in the Gcgr^*β-cell−/−*^ islets were unlikely to propagate across the islet, making the identification of a leading edge less applicable here. Therefore, the use of a FWHM measurement in these datasets, as a measure of the wave interval, results in slower estimation of wave propagation speeds, for both control and Gcgr^*β-cell−/−*^ islets, than reported by others [33,34]. A recent study found *in vitro* evidence for the critical importance of glucagon in the first-phase response of islets to a glucose stimulus. The first-phase GSIS response is conditional on cAMP signalling which, above a certain threshold, plays a permissive role for insulin secretion. Thus the paracrine effect of glucagon may be important for maintaining β-cell cAMP-tone [35] and, by extension, electrical coupling. Intriguingly, glucagonergic stimulation of islets has been shown to drive oscillatory cAMP activity [36,37] which synchronises with [Ca^2+^]_I_ responses in β-cells. Direct observation of islet [Ca^2+^]_I_ dynamics in this model reveals a smaller number of active cells at 10 mM glucose. We suggest that reduced cAMP tone in this model may reduce connectivity between β-cells resulting in reduced frequency of [Ca^2+^]_I_ waves. However, this seems to be offset by a longer duration of activity. Thus, overall, the genetic loss of the β-cell GCGR in this model results in a subtle dysglycaemic phenotype.

When transplanted into the anterior eye chamber of the mouse, islets become vascularised and receive innervation patterns homologous to those in the pancreas, acting as faithful “reporters” [38,39]. Around half of the Gcgr^*β-cell−/−*^ islets transplanted in the eye did not mount secretory wave activity, and in those that did, measures of β-cell connectivity were significantly reduced. In the *in vivo* environment the cAMP tone of β-cells may be supported by a multitude of other signals. For example, cholinergic receptor activation has also been shown to have synchronising properties in islets [40]. Deciphering which are the most important factors will help the development of novel treatments that restore co-ordinated insulin release. Given the differences in the topographical relationships of α- and β-cells across species, as well as differences in aspects of innervation, it will be important to investigate the relevance of these findings to human islets.

To better understand the impact of metabolic insults on β-cell [Ca^2+^]_I_ oscillations *in vivo*, we functionally assessed reporter islets longitudinally in the eye of DIO animals with a hyperglycaemic phenotype. The induction of DIO dysglycaemia caused a diminution in pan-islet [Ca^2+^]_I_ waves and β-cell connectivity read-outs, suggesting that high-fat feeding interferes with the ability of β-cells to propagate [Ca^2+^]_I_ across the islet. Whilst some have questioned the potential decoupling of insulin secretory activity from [Ca^2+^]_I_ dynamics with the use of certain anaesthetic agents (including isoflurane which was used in these experiments), we note that these *in vivo* findings mirror numerous previous *ex vivo* and *in vitro* reports of the deleterious effects of lipotoxicity on coordinated insulin secretory behaviour [[41]], [42], [43]]. It is worth noting that others have reported a rise in islet [Ca^2+^]_I_ wave amplitude during HFD, which the authors concluded was related to the hyperinsulinism and adaptive mechanisms that occur early on in the disease as a response to insulin resistance [44]. Do et al. found no change in the glucose [Ca^2+^]_I_ response as measured using ratiometric dyes in early diabetes but reduced pan islet [Ca^2+^]_I_ activity in advanced diabetes in db/db islets [45]. The loss of [Ca^2+^]_I_ wave propagation and the lower amplitudes when they did occur in our models may represent the fact that the animals were already frankly diabetic by 12 weeks on HFD. The precise mechanism by which HFD causes a loss of synchronised oscillations is presently unknown. However, it has been postulated that the deleterious effects of free fatty acids (FFAs) include suppressing the expression of gap junctions and reducing islet responsiveness to incretins [46].

In recent years, dual GLP-1R/GCGR agonists have gained increasing research attention due to their potential for superior weight-lowering effects and improved glycaemic control compared with GLP-1-based monotherapies [13,47]. As an example, cotadutide is currently being tested in Phase 2 clinical trials following its promising effects on DIO mice and non-human primates [48]. The additional weight loss evoked by cotadutide is attributed to GCGR-driven mechanisms to increase energy expenditure. Concurrently, GLP-1R agonism is thought to counterbalance any glucose-raising aspect of the glucagonergic element and to improve the glycaemic profile of DIO mice. However, after adjusting for weight loss, cotadutide-treated animals still show better glucose response curves than liraglutide-treated DIO mice [49]. We sought to investigate the direct effects of glucagon receptor activation in a clinically relevant model of obesity-associated hyperglycaemia, using a peptide agonist which was over ten times more potent at the glucagon than the GLP-1 receptor. Chronic treatment with this GCGR agonist for 40 days resulted in a markedly improved glycaemic profile in DIO mice. This improvement in glucose homoeostasis coincided with a 12 ± 2% weight loss, yet the restoration of β-cell connectivity and co-ordinated pan islet [Ca^2+^]_I_ activity measured with the GCGR-analogue was not recapitulated with equivalent weight loss alone, supporting the assertion that GCGR-activation promotes β-cell connectivity independently of weight loss. This raises the possibility that GCGR agonism may be particularly useful in restoring insulin secretory function in patients who are unable to lose enough pancreatic fat with dieting to resolve their T2D.

## 4 Future directions and conclusion

Pancreatic islets are micro-organs which integrate numerous neural, vascular and paracrine regulatory inputs to fine-tune their hormone secretory output to maintain glucose homoeostasis. In this study, we provide evidence for the role of intra-islet GCGR signalling in pan-islet [Ca^2+^]_I_ oscillations and first-phase insulin release *in vivo*. The phenotype of Gcgr^*β-cell−/−*^ animals is altered first-phase insulin secretory responses during an intravenous glucose challenge, redolent of pre-diabetes. Future studies should aim to fully elucidate the contributions of endocrine and paracrine signalling, gut hormone receptor distribution and function, to co-ordinated islet activity. This is necessary to guide future multi-agonist development for the treatment of T2D.

## 5 Methods

### 5.1 Animals

All procedures involving animals were conducted in accordance with the UK Animal (Scientific Procedures) Act 1986, under Project Licence PPL 75/0462. Gcgr^*fl/fl*^ mice on C57BL/6J background were generated by inserting a locus of X-over P1 (*LoxP*) site upstream of exon 6 on chromosome 11. A flippase recognition target (*FRT*) and *LoxP*-flanked neomycin resistance cassette was inserted downstream of exon 12. Animals with the neomycin resistance cassette were crossed with mice expressing flippase (*FLP*) to remove the *FRT*-insert, resulting in litters with *LoxP* sites at exons 6 and 12 of chromosome 11. For the β-cell specific deletion of the GCGR, Gcgr ^*fl/fl*^ animals were crossed with Ins1^Cre^-expressing animals (42). The Ins1^*Cre*^GCGR^*fl/fl*^ mouse line was bred in-house and produced normal litter sizes. Mice expressing the Ins1^*Cre*^ construct and homozygous for Gcgr ^*fl/fl*^ are referred to as ‘Gcgr ^*β-cell−/−*^’ within the text. Mice that do not express *Cre recombinase* under the Ins1 promoter (Ins1^*Cre*^–GCGR^*fl/fl*^) were used as controls and referred to as ‘littermate controls’ within the text.

For [Ca^2+^]_I_ imaging studies, Ins1^*Cre*^-expressing mice were crossed with mice that expressed GCaMP6f^*fl/fl*^ fluorescent calcium sensor, downstream of a *LoxP*-flanked STOP cassette (The Jackson Laboratory, stock no. 028865) and bred in-house. For [Ca^2+^]_I_ imaging studies with GCGR^*β-cell−/−*^ islets, the Ins1^*Cre*^GCGR^*fl/fl*^ mouse line was crossed with Ins1^*Cre*^–GCaMP6f^*fl/fl*^ mice for β-cell specific GCGR deletion and expression of the [Ca^2+^]_I_ sensor GCaMP6f. These mice are referred to as ‘GCaMP6f: Gcgr^*β-cell−/−*^’ within the text. Lastly, Ins1^*Cre*^GCaMP6f^*fl/fl*^ animals, with intact β-cell GCGR-signalling and the GCaMP6f calcium reporter, were used as controls to GCaMP6f: Gcgr^*β-cell−/−*^ islets and in the chronic GCG-analogue study.

For experiments with the GCG-analogue, Ins1^*Cre*^GCaMP6f^*fl/fl*^-expressing islets were transplanted into the anterior eye chamber of C57BL/6J syngeneic wild-type (WT) recipients (Envigo, Huntingdon UK). All animals were maintained under controlled conditions (21–23 °C; 12:12 h light:dark schedule) and following full implantation (>4 weeks) implanted islets (n = 3–4 per animal) were imaged: at baseline (normal chow), two months after HFD (5.21 kcal/g; 20% kcal protein, 60% kcal fat and 20% kcal carbohydrate; Research Diets, D12492) to induce DIO, and 40 days of daily sub-cutaneous injections of vehicle (saline + Zn^2+^; n = 8) or GCG-analogue (n = 8) and after weight-matching (n = 8) intervention. The synthetic GCG-analogue was administered subcutaneously. Metabolic tests (intraperitoneal glucose and insulin tolerance tests) were conducted at baseline, after HFD (to ascertain DIO) and after treatment.

### 5.2 qPCR Analysis

RNA from tissue samples was extracted using the guanidium thiocyanate-phenol-chloroform method. To quantify gene expression both probe-based (TaqMan® gene expression assay) and dye-based (SYBR® Green JumpStart™ Taq ReadyMix™ kit) methods were used. Reactions were performed in CFX384™ detection system (Bio-Rad).

### 5.3 Metabolic testing

Intraperitoneal glucose tolerance tests (IPGTTs) (2 g/kg), intraperitoneal insulin tolerance tests (IPTTs) (1 U/kg) and intraperitoneal pyruvate tolerance tests (IPPTTs) (2 g/kg) throughout this study were conducted after 5 h fasting. Blood glucose measurements were taken at −15, 0, 15, 30, 60 and 120 min time-points.

### 5.4 Hyperglycaemic clamp experiments

Under isoflurane anaesthesia (2%), indwelling catheters were surgically implanted into left common carotid artery and right jugular vein of Gcgr^*β-cell−/−*^ mice and littermate counterparts, and allowed to recover for 7 days. 50% glucose solution was continuously infused into the jugular vein while 10 U/ml heparinised saline was inserted into the carotid artery to withdraw blood samples during a hyperglycaemic clamp. GIR was measured over 45 min of plateaued glucose levels (typically achieved at 20 min after clamp start).

### 5.5 Insulin, glucagon and GLP-1 immunoassays

Hormones from blood samples of Gcgr^*β-cell−/−*^ mice and littermate controls were measured using standard ELISA kits (for insulin and glucagon, 62IN3PEF and 62SGLPEB, Cisbio, France; GLP-1, 81508, Crystal Chem, UK).

### 5.6 Dynamic *in vitro* glucose-stimulated insulin secretion experiments

Isolated islets from Ins1CreGcgr*^β-cell-/--^*expressing and control animals (n=3-4 animals per group; 50IEQ per experiment) were placed in a perifusion chamber (Biorep Technologies, Miami, FL.), pre-filled with a polyacrylamide P4 BioGel (Biorad, Hercules, CA.). Islets were perifused in Krebs-Ringer bicarbonate-HEPES (KRBH) (10mM HEPES, 2mM NaHCO_3_, 140mM NaCl, 3.6mM KCl, 0.5mM NaHPO_4_, 0.5mM MgSO_4_, 1.5mM CaCl_2_) solution supplemented with 0.25% (w/v) bovine serum albumin (BSA) at a pH of 7.4 with a glucose concentration of 3mM for 10min., then 10mM glucose KRBH for 20mins., followed by 20mM glucose KRBH for 20mins., and 40mM KCl KRBH for 10min., at a rate of 1mL/min. The effluent was collected at 1min. intervals and the insulin content of effluent samples was analysed using the CisBio HTRF Ultra-Sensitive Insulin kit (PerkinElmer) as per manufacturer`s suggestions instructions.

### 5.7 *In vitro* perifusion experiments

*In vitro* perifusion experiments were recorded using Yokogawa CSU22 Nipkow spinning disk microscope coupled with a Zeiss Axiovert M200 and x10/0.3NA objective (Zeiss) and Volocity software. Islet [Ca^2+^]_I_ dynamics (ex.:488nm; exp. time: 700msec) were recorded at 0.8Hz. In *in vitro* perifusion studies, Ins1*^Cre^*GCaMP6f*^fl/fl^*: Gcgr*^β-cell-/-^*-expressing (n=81 islets from 3 animals) and control (Ins1*^Cre^*GCaMP6f*^fl/fl^* Gcgr*^β-cell+/+^*) islets (n=63 islets from 4 animals);were first exposed to 3mM glucose KRBH with 1% (w/v) BSA for 10 minutes, then 10mM and 20mM glucose KRBH for 20 minutes each, followed by 10 minutes of 40mM KCl KRBH.

### 5.8 Wave characteristics

All waveform analyses were performed using a custom MATLAB script, available upon request. Briefly, relative amplitude was determined after normalizing [Ca^2+^]_I_ traces to F_min_. Frequency was defined as the total number of peaks over the time duration of a given glucose concentration. The full width at half maximum (FWHM) of a wave event was determined to express the time spent at half maximal [Ca^2+^]_I_ intensity.

### 5.9 Wave Propagation

A wave event was defined as a rise in normalised intracellular GCaMP fluorescence of 1.2 or above which covered an area of ≥30µm and returned to baseline GCaMP fluorescence values at the end of the cycle. The average velocity of wave events was determined as the distance covered between the start and end of a wave event over wave event duration. The average wave propagation ratio was calculated as the distance covered between the start and end of a wave event over total islet size (control group: n=140 events from 48 islets from 4 animals; Ins1*^Cre^*GCaMP6f*^fl/fl^*: Gcgr*^β-cell-/-^* group: n=90 events from 45 islets from 3 animals).

### 5.10 *In vitro* and *in vivo* connectivity

Single cell [Ca^2+^]_I_ traces were subjected to connectivity analysis as described in Salem et al. 2019.

### 5.11 Peptide generation and receptor activation (cAMP) assay

The GCG-analogue analogue was designed and donated by Professor SR Bloom (Imperial College London). Peptides were synthesised by Bachem (UK) and underwent local testing for purity after aliquoting and freeze drying, in the Bloom Drug Development programme. Cyclic AMP (cAMP) analysis was conducted using DiscoverX PathHunter cells expressing human GCGR, GLP-1R or GIPR using the cAMP Dynamic 2 cAMP kit (Cisbio, France) as per manufacturer's instructions.

### 5.12 Static *in vitro* glucose- and GCG-Ang-stimulated insulin secretion experiments

Isolated islets from WT animals (10IEQ per experiment) were first incubated in 3mM glucose KRBH, followed by a 1hr. incubation in either 12mM glucose or 12mM glucose + 20nM GCG-analogue KRBH solution. The insulin content of the supernatant was analysed using the CisBio HTRF Untra-Sensitive Insulin kit (PerkinElmer) as per manufacturer`s suggestions.

### 5.13 *In vivo* imaging experiments

Islets implanted in the anterior eye chamber were imaged (ex.:488 nm; at 3 Hz) under isoflurane anaesthesia (<2%) using a spinning disk confocal microscope (Nikon Eclipse Ti, Crest spinning disk, 20× water dipping 1.0 NA objective) under high circulating glucose.

### 5.14 Statistical analyses

Statistical significance between two conditions was assessed using the paired or unpaired, Student's *t*-test. Interactions between multiple conditions were determined using one- or two-way analysis of variance (ANOVA) (with Tukey's or Bonferroni's post-hoc tests). Analyses were performed using GraphPad Prism (GraphPad Software v.8.0) and MATLAB (Mathworks) and significant *p* values are described in each relevant section. Values are plotted as mean ± s.e.m., unless otherwise stated.

## CRediT authorship contribution statement

**K. Suba:** Writing – review & editing, Writing – original draft, Project administration, Investigation, Formal analysis, Data curation. **Y. Patel:** Investigation. **A. Martin-Alonso:** Investigation. **B. Hansen:** Writing – review & editing, Formal analysis. **X. Xu:** Formal analysis. **A. Roberts:** Investigation. **M. Norton:** Investigation. **P. Chung:** Investigation. **J. Shrewsbury:** Investigation, Formal analysis. **R. Kwok:** Investigation. **V. Kalogianni:** Investigation. **S.** **Chen:** Investigation. **X. Liu:** Investigation. **K. Kalyviotis:** Investigation, Formal analysis. **G.A. Rutter:** Writing – review & editing. **B. Jones:** Writing – review & editing, Formal analysis. **J. Minnion:** Writing – review & editing, Resources. **B.M. Owen:** Writing – review & editing. **P. Pantazis:** Writing – review & editing. **W. Distaso:** Writing – review & editing, Formal analysis. **D.J. Drucker:** Writing – review & editing, Conceptualization. **T.M. Tan:** Writing – review & editing. **S.R. Bloom:** Writing – review & editing. **K.G. Murphy:** Writing – review & editing, Methodology, Data curation, Conceptualization. **V. Salem:** Writing – original draft, Validation, Project administration, Methodology, Investigation, Funding acquisition, Formal analysis, Data curation, Conceptualization.

## Declaration of competing interest

All authors declare no conflicts of interest.

## Figures and Tables

**Figure:**
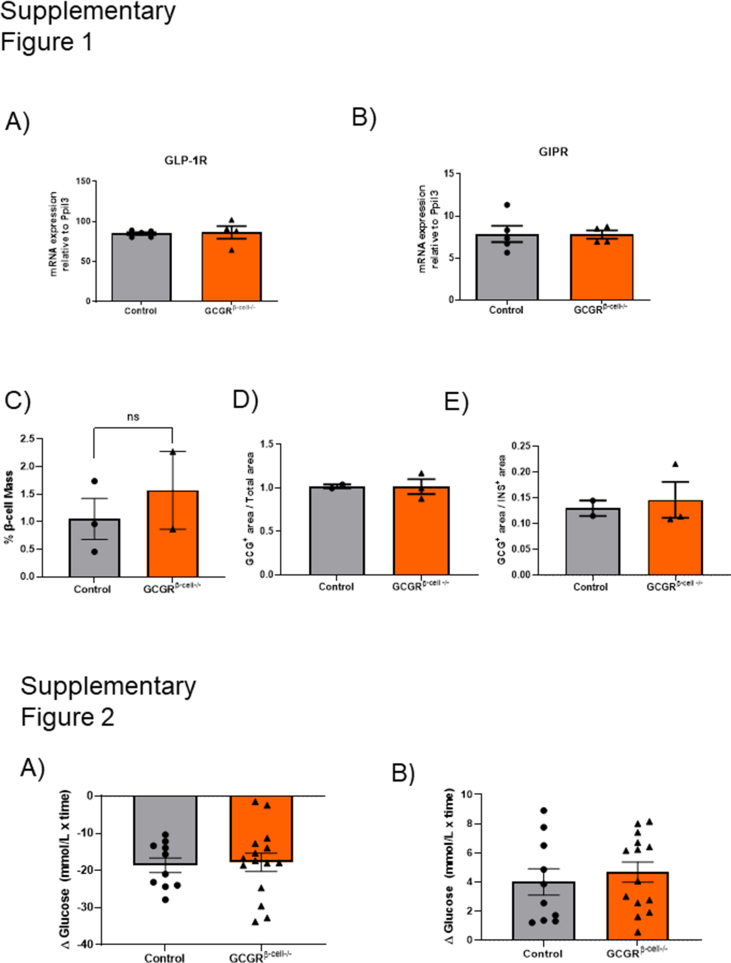
**Supplementary Figure 1**. GLP-1 and GIP receptor expression and the relative α- and β-cell content is unaltered in the islets of Gcgr^*β-cell−/−*^ animals. A) Relative GLP-1R expression levels were 84.9(±1.9) for controls versus 86.4 (±7.9) for Gcgr^*β-cell−/−*^ animals (Two-tailed, Mann–Whitney test; *p* = ns). B) Relative GIPR expression levels were 7.8(±1) for controls versus 7.8 (±0.5) for Gcgr^*β-cell−/−*^ animals (Two-tailed, Mann–Whitney test; *p* = ns). C) β-cell mass relative to total pancreatic tissue area is not significantly different in Gcgr^*β-cell−/−*^ (n = 2) versus control (n = 3) animals (Two-tailed, Mann–Whitney test; *p* = ns). D) α-cell mass relative to total pancreatic tissue area and E) α- to β-cell ratio is not significantly different in Gcgr^*β-cell−/−*^ (n = 3) versus control animals (n = 2; Two-tailed, Mann–Whitney test; *p* = ns). **Supplementary Figure 2**. There is no difference in glucose excursion following insulin or pyruvate tolerance tests between Gcgr^*β-cell−/−*^ and littermate control animals. Insulin (A) and pyruvate (B) tolerance testing is unaltered in Gcgr^*β-cell−/−*^ animals (n = 15) compared to controls (n = 10; Two-tailed, Unpaired t-tests and Mann–Whitney test; *p* = ns).

**Figure:**
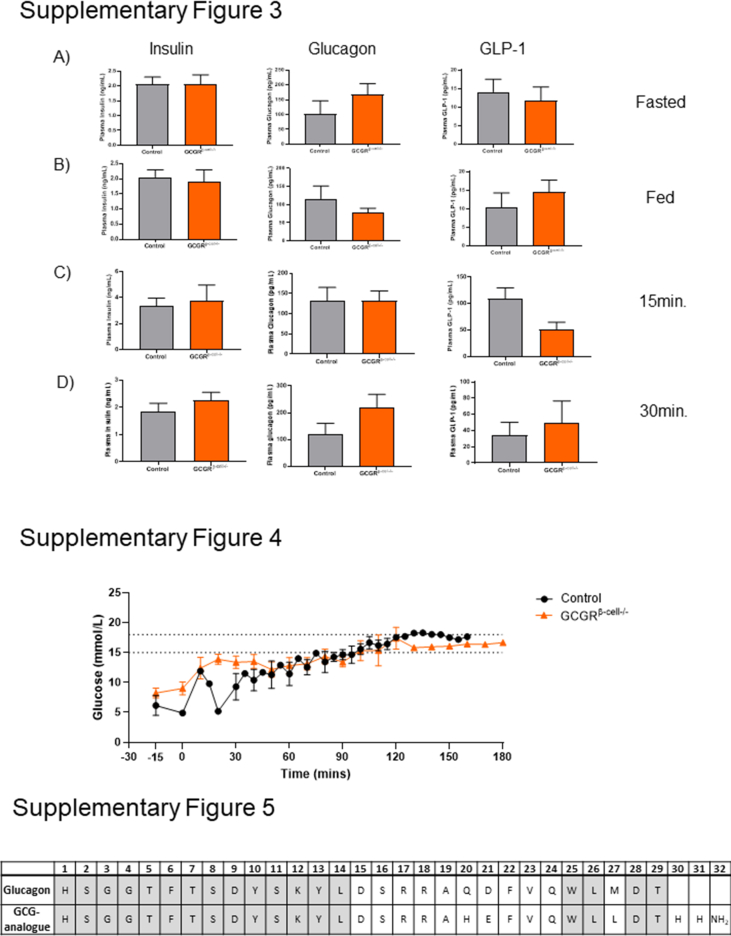
**Supplementary Figure 3**. Serum hormone levels during an IPGTT are comparable Gcgr^*β-cell−/−*^ and control animals. Insulin, glucagon and GLP-1 levels in A) fasted, B) non-fasted, C) at 15 min. and D) 30 min. during an IPGTT are comparable between control and Gcgr^*β-cell−/−*^ animals (two-tailed, Unpaired t-test of timed measurements; *p* = ns). **Supplementary Figure 4**. Glucose curves from hyperglycaemic clamp study in Gcgr^*β-cell−/−*^ and littermate control animals. **Supplementary Figure 5**. The synthetic GCG-analogue. A synthetic analogue of native glucagon was generated with amino acid changes in positions 21 (from aspartate to glutamate) and 27 (from methionine to leucine) and with a further addition of two histamine groups preceding an amine group.

**Figure:**
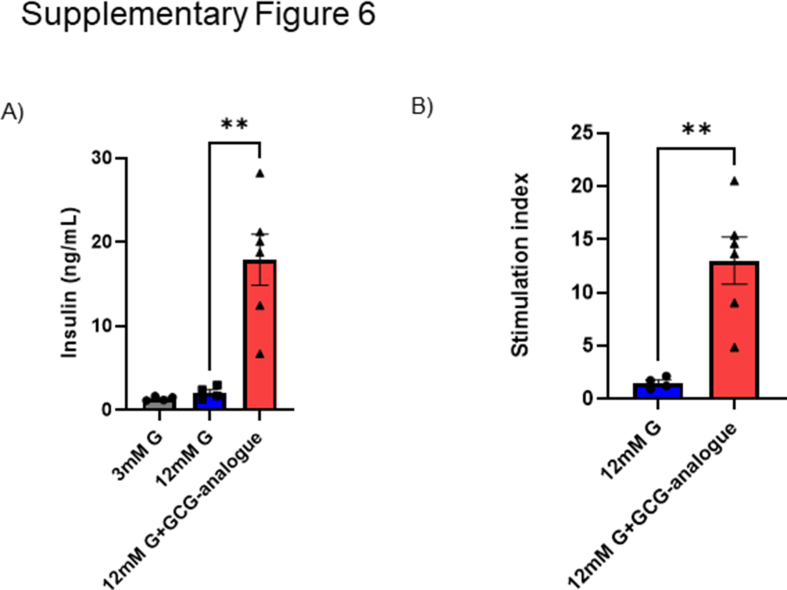
**Supplementary Figure 6**. The GCG-analogue stimulates insulin secretion from WT islets *in vitro*. A) Absolute levels of insulin secreted from isolated WT islets (60 islets from 3 animals; 10IEQ per assay) is significantly elevated in the presence of the GCG-analogue (2.08 ± 0.37 ng/mL versus 17.92 ± 3 ng/mL for 12 mM glucose versus 12 mM glucose + GCG-analogue, respectively; one-way ANOVA; ∗∗*p* = 0.0015). B) Similarly, the stimulation index of WT islets is higher in the presence of the GCG-analogue (unpaired t-test; ∗∗*p* = 0.003).

## Data Availability

Data will be made available on request.
